# A Proposal for a Scientifically-Informed and Instrumentalist Account of Free Will and Voluntary Action

**DOI:** 10.3389/fpsyg.2017.00754

**Published:** 2017-05-17

**Authors:** Eric Racine

**Affiliations:** ^1^Neuroethics Research Unit, Institut de recherches cliniques de MontréalMontréal, QC, Canada; ^2^Department of Medicine and Department of Social and Preventive Medicine, Université de MontréalMontréal, QC, Canada; ^3^Department of Neurology and Neurosurgery, Experimental Medicine and Biomedical Ethics Unit, McGill UniversityMontréal, QC, Canada

**Keywords:** free will, voluntary action, autonomy, ethics, pragmatism, neuroscience

## Abstract

The ability to choose freely is captured under the umbrella concept of “free will,” which designates an ability that plays a crucial role in most understandings of autonomy and responsibility and, thus, bears significance for moral practice and moral theory. Some claim that neuroscience research challenges the existence of free will/voluntary action while some who adopt stronger eliminativist stances have gone as far as describing free will as an illusion. Contrary to that, those relying on realist stances have restated the foundational value and role of folk psychological concepts of voluntary action and free will in, for example, the domains of ethics and law. An emerging body of research in cognitive science and social psychology has generated results suggesting that the phenomena captured by the concepts describing free will and voluntary action are dynamic and responsive to priming and framing effects. We propose that this body of research suggests the existence of dynamic and consequential properties of free will better captured following pragmatist theory and instrumentalist epistemology. This contrasts the simpler static concept of free will and the related metaphysics that was at the basis of earlier debates and structured around the poles of realism and eliminativism. This paper contextualizes ontological and epistemological debates about free will, describes a scientifically-informed and instrumentalist account of the concept of free will and voluntary action consistent with recent research in cognitive science, and discusses its implications for research (e.g., theoretical assumptions of research paradigms, interdisciplinary research) and practice (e.g., impact on self-image and social behavior).

The approach to psychological theory from the standpoint of the organism must inevitably be through an emphasis upon conduct, upon the dynamic rather than the static.

—George Herbert Mead, *Mind, Self*, & *Society*, (Mead, 1934)

## Background

The ability to choose freely and without constraints is commonly captured by the concept of “free will” (FW) although there is significant fuzziness surrounding this concept (Roskies, 2006). A more concrete and meaningful rendition of the concept could be the “ability to do otherwise” (Baumeister and Monroe, 2014). Neuroscience and cognitive science have now investigated the ability to initiate conscious and voluntary action—often but not always designated as “free will”—through a broad range of studies and contributions such as electroencephalography (EEG), functional Magnetic Resonance Imaging (fMRI Libet et al., 1982, 1983; Haggard et al., 2003; Bode et al., 2011), and vignette-based and priming studies (Stroessner and Green, 1990; Vohs and Schooler, 2008; Pronin and Kugler, 2010; Ent and Baumeister, 2014). These studies have reinvigorated a long and very rich tradition of discussions on the nature and existence of FW and voluntary action (VA) (Dilman, 1999), including metaphysical discussions about FW, i.e., ontological debates on the existence of FW, and its alleged profound concrete and conceptual implications. At a very basic level, these discussions revolve around whether moral agents have the fundamental ability to engage in conscious, deliberate choices or actions or whether moral agents are under the influence, even the control, of mechanisms (e.g., non-conscious, “automatic,” events, or processes) that question the ability of the moral agent to initiate voluntary actions (Smith, 2011). Of course, such debates relate directly to the fundamental concepts of autonomy and responsibility in ethics, for which voluntary action is crucial (Felsen and Reiner, 2011; Dubljević, 2013). Indeed, if the ability of moral agents to act is jeopardized or if agents lack the ability to initiate actions which can be qualified of conscious and deliberate, then neither can they be faulted for their own actions (responsibility) nor encouraged to undertake actions on the premise of their own preferences (autonomy).

Another separate debate is sparked by recent research in cognitive science and social psychology that suggests that the concept of FW/VA^1^ may describe a psychological phenomenon^2^ which has intriguing dynamic and consequential properties. The dynamic properties of FW/VA designate possible changes to FW/VA in response to the internal (physiological) and external (physical and social) environment. For example, research suggests that the phenomenon of FW/VA is not static but shaped by felt physiological needs or deterministic prompts (Rigoni et al., 2011, 2012, 2013, 2015). The consequential properties of FW/VA refer to the fact that changes in FW/VA can have implications such as the finding that diminished FW can lead to unethical behavior like cheating (Vohs and Schooler, 2008) while increased BFW predicts pro-social behavior and work performance (Baumeister et al., 2009; Stillman et al., 2010). These dynamic and consequential properties of the phenomenon of FW/VA could potentially challenge the initial academic impetus and legitimacy to confirm or invalidate the existence of FW/VA *per se*, at least in the strong terms of prior ontological and metaphysical debates. Indeed, these properties suggest that free will is a capacity of the agent whose ontology stems from a very real agential and first-person perspective consistent with research on folk views on FW supporting the psychological reality of the phenomenon (Nahmias et al., 2004, 2005, 2007; Nahmias and Murray, 2010; Stillman et al., 2011; Baumeister and Monroe, 2014). This idea of the first-person ontology of FW/VA it not novel (see Gert and Duggan, 1979) but recent research brings empirical support for the psychological dimension of FW and how questions about FW must recognize its first-person origins. Because it drives powerfully home this idea, recent research also brings important theoretical and practical questions about what this research contributes and what it could change, practically speaking, if the concept of FW is based on a better understanding of FW as a psychological phenomenon.

In this paper, I explore implications of recent research in cognitive science and social psychology on the phenomenon of FW/VA based on previous similar suggestions which have hinted at a distinct contribution of psychology to this debate (Baumeister, 2008) although so far there has been hesitation to draw more philosophical conclusions from this work. I argue that much of the previous conversation about free will has been structured around an ontological debate about eliminativism and realism where the existence of FW/VA has been the central issue and where the concept of FW/VA entertained has been describing a largely static phenomenon. In contrast, recent research in cognitive science and social psychology brings another conversation to the forefront, one which is more epistemological in nature and brings to light the psychological properties of the phenomenon of FW/VA. After contrasting briefly these two bodies of scholarship, I argue that the more recent contribution of neuroscience and cognitive science carries some potentially important transformative implications for research and practice not well-captured thus far. Overall, the recognition of the dynamic and consequential properties of the phenomenon of FW/VA calls for nothing short of a paradigm shift in academic inquiry. I suggest we replace the initial static ontological and metaphysical framework of FW/VA with a dynamic epistemological framework. This shift is supported by recent (1) social psychology and cognitive science research showing how important the psychological phenomenon of FW is because of its consequential and dynamic properties as well as (2) pragmatist philosophy (e.g., writings from Dewey, Mead, and Dennett) that supports an instrumentalist analysis of the concept of FW. This instrumentalist account recognizes the properties of FW put to light by recent scientific research and fits within a broader dynamic epistemological framework inspired by pragmatism where synergetic inquiries offer path forward for research. The notion of synergetic inquiries refers here to how the first-person perspective on FW/VA is enriched by the results of scientific investigations and to how scientific investigations are grounded in the psychological reality of the phenomenon of FW. (The result of such synergetic investigations is described as resulting in “synergetic enrichment” or “synergetically enriched” concepts.) Consistent with the philosophy of pragmatism, the proposed paradigm shift involves deconstructing the implied assumptions found in “common-sense” understandings of FW through critical reflection and experimental research—including those at the basis of philosophical discussions—and also to then refurnish them with enriched meaning based on scientific inquiry on the nature of the phenomenon of FW and critical reflection.

## The philosophical implications of scientific investigations on free will/voluntary action

From the perspective of applied ethics and social behavior, voluntariness is a key dimension in the understanding of autonomous decisions and actions as well as our responsibility toward and ownership of these decisions and actions (Dworkin, 1988; Wegner, 2002). Autonomous decisions and actions imply that the agent is initiating them according to his or her own wishes and that the person is free to do so (i.e., not under direct or indirect forms of coercion that would imperil the existence of such an ability). Accordingly, in applied ethics, voluntariness commonly refers to “the degree that [the moral agent] wills the action without being under the control of another's influence” (Beauchamp and Childress, 2001). Indeed, if moral agents have a jeopardized ability, or even lack the ability to initiate actions freely, then neither can they be faulted for their own actions (responsibility) nor encouraged to undertake actions on the premise of their expression of their own preferences (autonomy; Felsen and Reiner, 2011; Castelo et al., 2012). The concept of FW commonly captures a basic form of agency and a responsibility associated with this ability to self-control and initiate voluntary action (Roskies, 2006; Brass et al., 2013). Accordingly, in this paper, FW designates primarily a basic ability to envision options and choose between them such that the will or volition of the person is considered to be free. In contrast, freedom designates a higher level capacity to not only envision actions but carry them out. Autonomy designates an even higher level and more sophisticated capacity to undertake actions freely and in ways which are consistent with one's values or preferences (Racine and Dubljević, 2017). Autonomy requires freedom, which itself requires free will, but free will does not necessarily entail freedom, and freedom does not necessarily lead to autonomy. Otherwise said, having a basic ability to envision options and choose would not entail that one has the freedom to actually carry out this choice (Racine and Dubljević, 2017). Obviously, the capacity for an agent to act in the world could be contingent on affordances of objects and social situations and not simply the will of the agent (Gibson, 1979). Accordingly, scientific investigations about FW/VA have significant philosophical import because they can enrich our understanding of FW/VA.

To date, two distinct, somewhat interconnected, strands of scientific research on FW/VA have surfaced: a first strand stems from neuroscience and is often embedded in an ontological conversation about the concept of FW. The second strand, stemming from social psychology and cognitive science, sets the stage for a more epistemological conversation about the dynamic and consequential properties of FW/VA and the synergetic enrichment of that concept based on such scientific observations.^3^

### First strand: neuroscience and the ontological debate about free will and voluntary action

There have been continuous debates in the philosophical tradition about the ability of individuals to manifest and exercise FW (Dilman, 1999). The key question has been whether humans *have* FW or not. Answers to this question have been categorized along a spectrum between the prototypical stances of “compatibilism” and “incompatibilism” regarding the ontology of FW/VA (see Table 1). Compatibilism captures theories that grant the possibility of the existence of FW, despite causal determinism. Incompatibilism designates theories which deny the existence of FW given the overriding domination of causal processes (or even deny the co-existence of FW and causal processes), which do not leave any room for FW.^4^

**Table 1:** **Typical stances on the contribution of neuroscience to the understanding of voluntary action and “free will.”**.

**Stances on contribution of neuroscience**	**Stances on the ontology of FW/VA**	
	**Exists (compatibilism)**	**Does not exist (incompatibilism)**
*Significant*	FW/VA is a crucial concept (describes a reality) and neuroscience will significantly inform or transform this concept.	FW/VA is a useless/flawed/imperfect concept and neuroscience will replace it with a more scientific concept.
*Insignificant*	FW/VA is a crucial concept (describes a reality) but neuroscience will have no meaningful impact on our understanding or use of this concept.	FW/VA is a useless/flawed/imperfect concept and neuroscience will not show anything meaningful and relatable to such (an ill-defined) concept.

*Stances on the ontology of FW/VA are distinct from stances on the contribution of neuroscience. The specific associations between these two separate issues are not logically necessary, contrary to some interpretations, i.e., that neuroscience can only invalidate the existence of FW/VA. I acknowledge that between these strong types, a spectrum exists in the positions identified. FW/VA = Free will/voluntary action*.

The debate about the existence of FW/VA pre-dates modern neuroscience but neuroscience research has reinvigorated ontological debates about FW. This began with a series of experiments conducted by Benjamin Libet in the 1980s. Libet investigated the “readiness potential,” i.e., brain activation prior to the initiation of a conscious and deliberate action. Using electroencephalography (EEG), which measures electric activity of neurons, he established the existence of a readiness potential prior to the subjects' own consciousness of having made a decision. In other words, the brain was shown to be active before the subjects were aware of their desire to initiate an action. It is beyond this short background to review the methodological and epistemological issues that challenge Libet's initial conclusions (Mele, 2009), but these experiments led Libet to propose that

“other relatively ‘spontaneous’ voluntary acts, performed without conscious deliberation or planning, may also be initiated by cerebral activities proceeding unconsciously. These considerations would appear to introduce certain constraints on the potential of the individual for exerting conscious initiation and control over his [this person's] voluntary acts.” (Libet et al., 1983)

Libet did grant that there

“would remain at least two types of conditions in which conscious control could be operative. (1) there could be a conscious ‘veto’ that aborts the performance even of the type of ‘spontaneous’ self-initiated act under study here […] (2) In those voluntary actions that are not ‘spontaneous’ and quickly performed, that is, in those in which conscious deliberation (or whether to act or of what alternative choice of action to take) precedes the act, the possibilities for conscious initiation and control would not be excluded by the present evidence” (Libet et al., 1983).

Libet's experiments and its legacy led to diametrically opposed interpretations, with few siding with his own original middle-ground interpretation supporting the existence of a veto power or a “free won”t' (Dubljević et al., 2014) although for an-example describing the significance of inhibition to support flexible behavior, see Mirabella (2014).^5^ In the most radical interpretations, the claim that free will is an illusion supports the notion that humans are not responsible for their actions, lack decisional capacity, and should not be held accountable for consequences of their actions. In this vein, Greene and Cohen have suggested that the notions of moral and legal responsibility would need to be extensively revised because the actions of moral agents are, they argue, largely determined (Greene and Cohen, 2004). They recommend moving away from retribution and support rehabilitation approaches in the criminal judicial system based on consequentialist moral theories. Based on Libet's initial findings and subsequent research, researchers have also claimed that FW is simply an illusion (Wegner, 2002). Others have followed Libet's lead to undertake a series of studies on the automaticity and unconscious components of behavior (Haggard et al., 2004; Haggard, 2005, 2011; Smith, 2011). These studies are sometimes interpreted as showing that FW does not exist because human behavior is essentially determined such that “[w]e feel we choose, but we don't” (Smith, 2011).

Contrary to that, some contend that the experiments and similarly-minded subsequent work in neuroscience does not threaten folk psychological beliefs in FW or VA (Roskies, 2006). For example, the behaviorist and legal scholar, Steven Morse, has argued that the strong incompatibilist interpretations rely on a “brain overclaim syndrome.” According to this view, neuroscience research bears no impact for the law because of the genuine value and usefulness of beliefs in responsibility and autonomous decisions as well as the rather abstract nature of FW (Morse, 2006, 2008). In ethics, Felsen and Reiner have proposed that a standard model of autonomy—defined on the criteria of rational deliberation, reflexive agency, and freedom from external influences (Felsen and Reiner, 2011)—is inconsistent with evidence emerging from neuroscience research, notably because of the implicit mechanisms and biases to which human thinking is prone. This suggests that the commonly assumed ability of moral agents to truly deliberate and be conscious about most actions undertaken is exaggerated. However, Felsen and Reiner have resisted concluding that moral agents cannot undertake autonomous actions and should not be held responsible for their actions because their will would be fundamentally constrained.

In spite of the divide between these opposed families of interpretations, both realists and eliminativists, have largely relied on common ontological beliefs that support such a debate, namely that free will can be examined from the third-person standpoint and that it represents a rather static phenomenon which has been essentialized and abstracted from social context and psychological reality for the sake of philosophical analysis (Racine and Saigle, 2014). This underlying set of static metaphysical assumptions, shared to various degrees, may have led to the neglect of the psychological reality of FW/VA (Table 1). Furthermore, the belief that neuroscience bears on the existence or inexistence of FW/VA integrates (and potentially conflates) two distinct types of claims: those regarding the existence of FW/VA and those regarding the impact of neuroscience on FW/VA on stances about the existence of FW/VA (see Table 1). These claims are not necessarily tied together when evaluated from both a conceptual (Dubljević, 2013) and an empirical standpoint given, for example, the findings that beliefs in determinism do not conflict with beliefs in FW in folk intuitions (Nahmias et al., 2014). Only of late, has research disentangled these two different sets of claims, thus making possible the scientific investigation of the nature of FW and beliefs in FW without being necessarily bound to metaphysical debates about the existence or inexistence of FW. Brass, Lynn, Demanet, and Rigoni write that, “only recently have researchers on the aforementioned fields [cognitive neuroscience, social psychology] come to realize that it might be useful to investigate willed behavior without trying to determine whether free will exists or not” (Brass et al., 2013). As such, it is important to note that the use of the concept of FW to designate a psychological phenomenon, one which stems from subjective and intersubjective experience, does not equate to the claim that FW does not exist. To get there, an additional argument would need to be made to the effect that concepts capturing subjective experience are somehow inherently vacuous, for example, because this concept designates a phenomenon which should not be considered to exist (because it is an illusion or otherwise very-ill defined).

### Second strand: cognitive science, social psychology, and the epistemological debate about the concept of free will and voluntary action

Neuroscience research has not only reinitiated an ontological debate about FW/VA but it has recently brought, notably with the additional contributions of cognitive science and social psychology, additional insights into the nature of the phenomenon of FW/VA, thus seeding the potential for a paradigm shift in research (Baumeister, 2008). This research involves a series of empirical studies stemming from cognitive science and social psychology which have examined the impact of belief in FW on actual behavior or, in some cases, the impact of behavior on FW/VA (Baumeister, 2008). For example, one iconic study showed that when presented with texts that encouraged a belief in determinism, participants were more likely to cheat on a self-reward task than those who were prompted with a text reflecting a belief in FW (Vohs and Schooler, 2008). Another study showed that when participants read phrases that discouraged a belief in FW, they were less helpful and were more aggressive (Baumeister et al., 2009). In an adaptation of Libet's original methodology (Libet et al., 1983) that introduced participants to different priming texts, participants who read information undermining FW had smaller readiness potentials (Rigoni et al., 2011). Rigoni and colleagues state that “the readiness potential was reduced in individuals induced to disbelieve in free will,” thereby indicating that “abstract belief systems might have a much more fundamental effect than previously thought” (Rigoni et al., 2011). The authors underscored the relationship between free will and behavior:

“[p]utting less effort into an action might weaken our sense of agency for these actions and lead to a reduced feeling of responsibility. This reduced feeling of responsibility would very likely result in more careless and irresponsible behavior. The basic assumption of this explanation is that disbelief in free will influences people's sense of agency” (Rigoni et al., 2011)

A more recent study inspired by literature on embodied cognition has concluded that the salience of basic physiological signals (e.g., urge to urinate, felt sexual desire) decreases FW (Ent and Baumeister, 2014). An interesting vignette-based study explored the association between neuroscience's ability to understand and predict behavior and concluded that this ability did not significantly impact lay beliefs in FW; rather beliefs in FW were imperiled when the efficacious nature of reasons in behavior were threatened (e.g., through manipulation; Nahmias et al., 2014). For example, when the efficacy of a mental state (an intent) is threatened by some form of manipulation, beliefs in FW diminish.

In sum, these recent studies suggest that the concept of “free will” captures a first-person experience of agency and that the concept of FW/VA is derivative of this experience. Accordingly, FW is a concept that designates a dynamic and consequential psychological phenomenon, a phenomenon which is influenced by personal factors and social contexts (Racine and Saigle, 2014). This assumption is rather opposed to the essentialism encountered in the first wave of discussion about the implications of neuroscience which relied on a more static concept of FW/VA, far removed from the outlook of the psychological sciences (Baumeister, 2008; see Table 2). Interestingly, the static, third-person, and essentialist aspect of the metaphysical concept of FW was criticized by Dewey as a philosophical Holy Grail. Dewey wrote with much despair that “[w]hat men have esteemed and fought for in the name of liberty is varied and complex—but [it] certainly has never been a metaphysical freedom of will” (Dewey, 1922). Dewey redefines free will largely as effective VA, i.e., the ability to carry out plans, the capacity to change them, and the power of the individual to be an actor in the course of events (Dewey, 1922). This in itself constitutes a relevant criticism of the underlying assumptions of ongoing ontological debate and its resistance to the incorporation of psychological and cognitive science. Dewey's critique is grounded partly in instrumentalism and in an analysis of the dead ends produced by static philosophical scholasticism as well as an absence of commitment to scientific inquiry as a source of knowledge to enrich such key concepts. What is surprisingly contemporary about this analysis is that some key neuroscience-derived messages about free will (even if Dewey would have applauded the input of neuroscience) have remained stuck in an overarching static metaphysical framework. This framework wrestles with the language of essences and the dualisms it generates as well as resulting problems such as the existence of an uncaused causer, mental causation (Kane, 2011) and other impressive philosophic-semantic puzzles. As a result, so far, the implications of research in psychology and cognitive science have remained rather undefined with respect to the future of scientific research in this area as well as to its practical implications (e.g., with respect to self-image, self-reflection, self-awareness of factors influencing one's sense of FW). Indeed, some leading researchers, have explicitly attempted to shield recent cognitive research from such discussions. For example, MacKenzie, Vohs, and Baumeister, write that “[g]rand questions about free will have prompted experts to engage in complex metaphysical, semantic, and even theological debates. Our work has nothing to say about these” (MacKenzie et al., 2014). Although this claim is understandable, and there is wisdom in respecting the empirical nature of much recent psychological research on FW, the next section of this paper attempts to bring this literature to bear on the debates about the nature of FW/VA. In doing so, I put forward a proposal for an instrumentalist analysis of FW/VA supported by recent research and with some possible practical implications. I contrast this instrumentalist account to an ontological and metaphysical concept of FW.

**Table 2:** Comparison of essentialist and instrumentalist accounts of the concept of FW/VA.^*^.

**Essentialist concept of FW/VA**	**Instrumentalist concept of FW/VA**
Concept of FW designates an all or none (dichotomous) property or state of agents or their decisions	A folk psychological and commonly used approximate and umbrella concept capturing voluntariness of action on a continuum
Static, does not change; refers to a fix state	Dynamic, can increase or decrease depending on one's cognitive or physical state
Essentially a third person concept, whose existence is confirmed or invalidated by science (science chiefly revises ontology)	Originates from one's own sense of agency, with respect to situations and with respect to one's own self-understanding and behaviors; an inter-subjective concept rooted in first person ontology, which can be understood through scientific inquiry (science chiefly revises epistemology)

* *The essentialist strategy emphasizes the search for the “true” nature of a phenomenon. Historically, the Western philosophical tradition has stressed that those essences would be fix, immutable, and universal. Dewey described this quest as the “philosophical fallacy” and suggested, following Peirce's work in logic, to define concepts in terms of their functional roles. Dewey referred to this approach as “instrumentalism” (Misak, 2013)*.

## From essentialist to instrumentalist accounts of free will and voluntary action

FW/VA has mostly been discussed in an ontological intellectual context where the key question was whether FW existed or not. In contrast, adequate recognition of the dynamic and consequential aspects of FW/VA in real world situations dictates an appropriately dynamic epistemological paradigm, which I will explore in three points: (1) we can attend to the dynamic and consequential properties of the phenomenon of FW/VA as it is augmented or diminished by experience and knowledge; (2) scientific knowledge (broadly construed) can offer “synergetic enrichment” between the phenomenon of FW/VA and the concept of FW/VA; (3) practical implications could eventually stem from a significantly revised understanding of FW/VA. I refer to this account of FW/VA as instrumentalist, aligned with Dewey's (and other pragmatists') instrumentalism calling for a critical analysis of the function and utility of concepts like FW/VA in daily life and the retooling of those concepts based on the contribution of dedicated scientific inquiries (Misak, 2013). This instrumentalism also has some affinities, to a lesser extent, with Dennett's treatment of psychological concepts in the “intentional stance” (Dennett, 1981) although like Dennett, Dewey also refers to folk psychology as being approximate and based on self-interpretation. Most importantly, my instrumentalism entails a movement away from essentialist debates about the static character of FW/VA and its existence.

### FW/VA has dynamic and consequential properties, FW/VA can be augmented, or diminished by experience and knowledge

**Figure 1:**
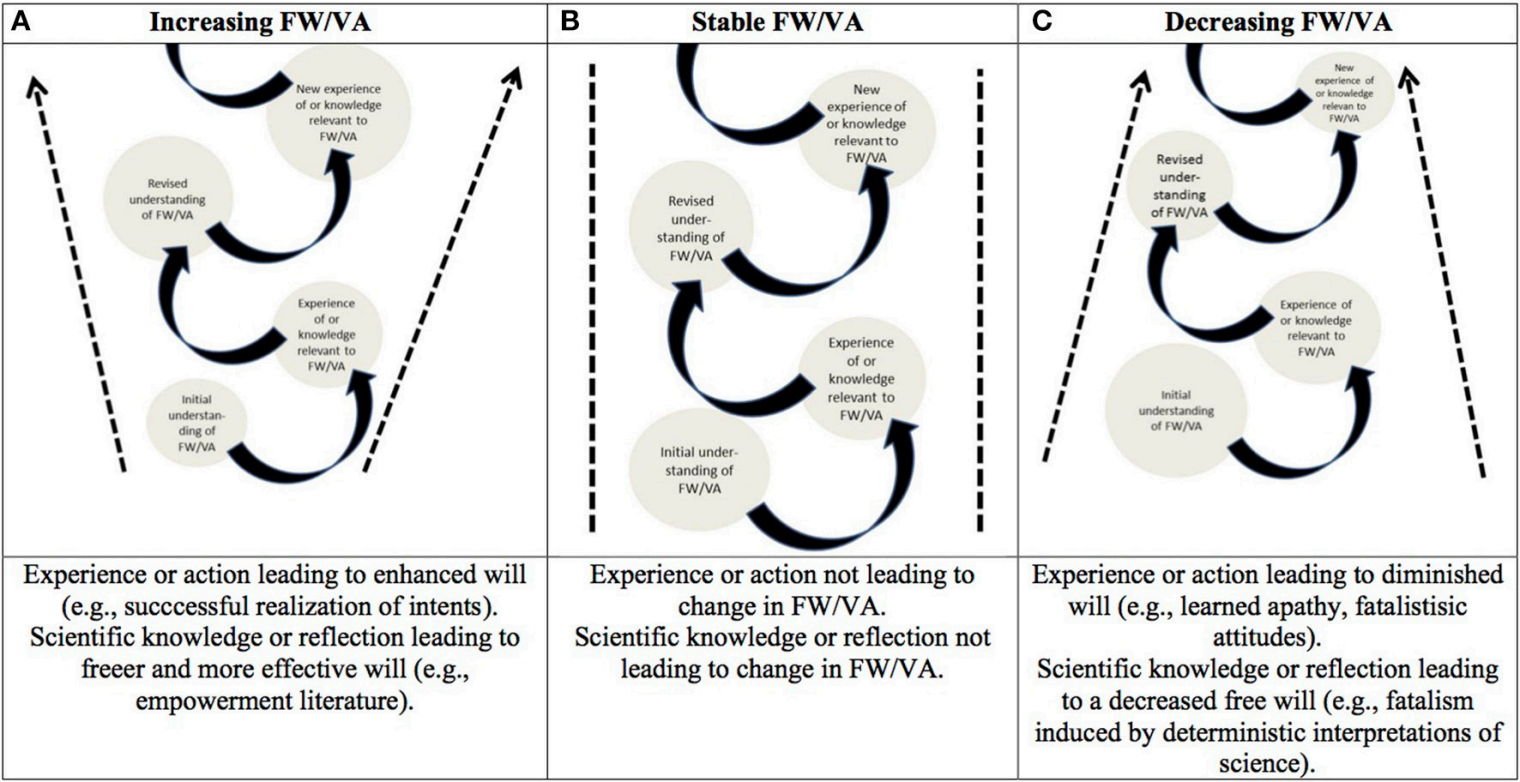
**Dynamic properties of the phenomenon of FW/VA and revised understandings of FW/VA FW /VA can be expanded or diminished by experience and knowledge**. The figure depicts different possible life movements of the phenomenon and concept of FW/VA. See text explanation. The porous conceptual domain depicted by the doted arrows represents the fact that in spite of the movement, there is some stability of the phenomenon and concept of FW/VA (they are not arbitrary and refer to subjective and intersubjective life experience).

The instrumentalist account of FW recognizes the dynamic and consequential aspects of FW/VA. Accordingly, three basic types of movements can occur: FW/VA can increase (Figure 1A); FW/VA can remain stable (Figure 1B); FW/VA can decrease (Figure 1C). These changes can occur either because of the contributions of (1) experience and action; and/or (2) scientific knowledge and self-reflection. For example, FW/VA can be expanded by experience and action given the successful completion of a willed action. It can also be enhanced by scientific knowledge that affects the will as an object [e.g., scientific knowledge or technology making possible an action that was otherwise impossible or difficult such as a brain-computer interface (Glannon, 2014)] or from a subjective standpoint (e.g., self-reflection leading to the empowerment of the moral agent). In contrast, the will can diminish based on experience and (lack of) action (e.g., inability to complete an action, inability to cease an addictive behavior). Scientific knowledge that informs about the will as an object (e.g., knowledge about the conditions or about lack of means that make an action less possible) or the will from a subjective standpoint (e.g., self-reflection leading to stronger beliefs in determinism) can reduce one's ability to initiate voluntary actions. It is clear that individual lives include a series of different scenarios and a range of experiences of (increasing/stable/diminishing) FW/VA based on contingencies of life, pathologies (e.g., depression, addiction), and one's interpretation of the situation (e.g., reinterpreting liberal economic market forces as a form of alienation of one's moral agency or a system in which one can pursue his or her own preferences). Importantly, changes in FW induce consequences and can have implications with respect to, for example, social, and moral behavior (Vohs and Schooler, 2008); otherwise this phenomenon would be of lesser interest.

This preceding analysis is consistent with instrumentalism in philosophy of mind as developed by Dewey and Dennett where the value of a folk psychological concept is contingent on its ability to explain and predict; leaving aside the debate about its ontology. Without being arbitrary, FW/VA is fluid and the concept of FW/VA is best considered a quintessence of self-interpretation, i.e., a good common sense approximation of complex psychological and behavioral phenomena. Indeed, Dewey suggests that, “the meanings of such words as soul, mind, self, unity, even body, are hardly more than condensed epitomes of mankind's age-long efforts at interpretation of its experience” (Gouintlock, 2002).

### Scientific knowledge (broadly construed) can offer “synergetic enrichment” between the phenomenon of FW/VA and the concept of FW/VA

**Figure 2:**
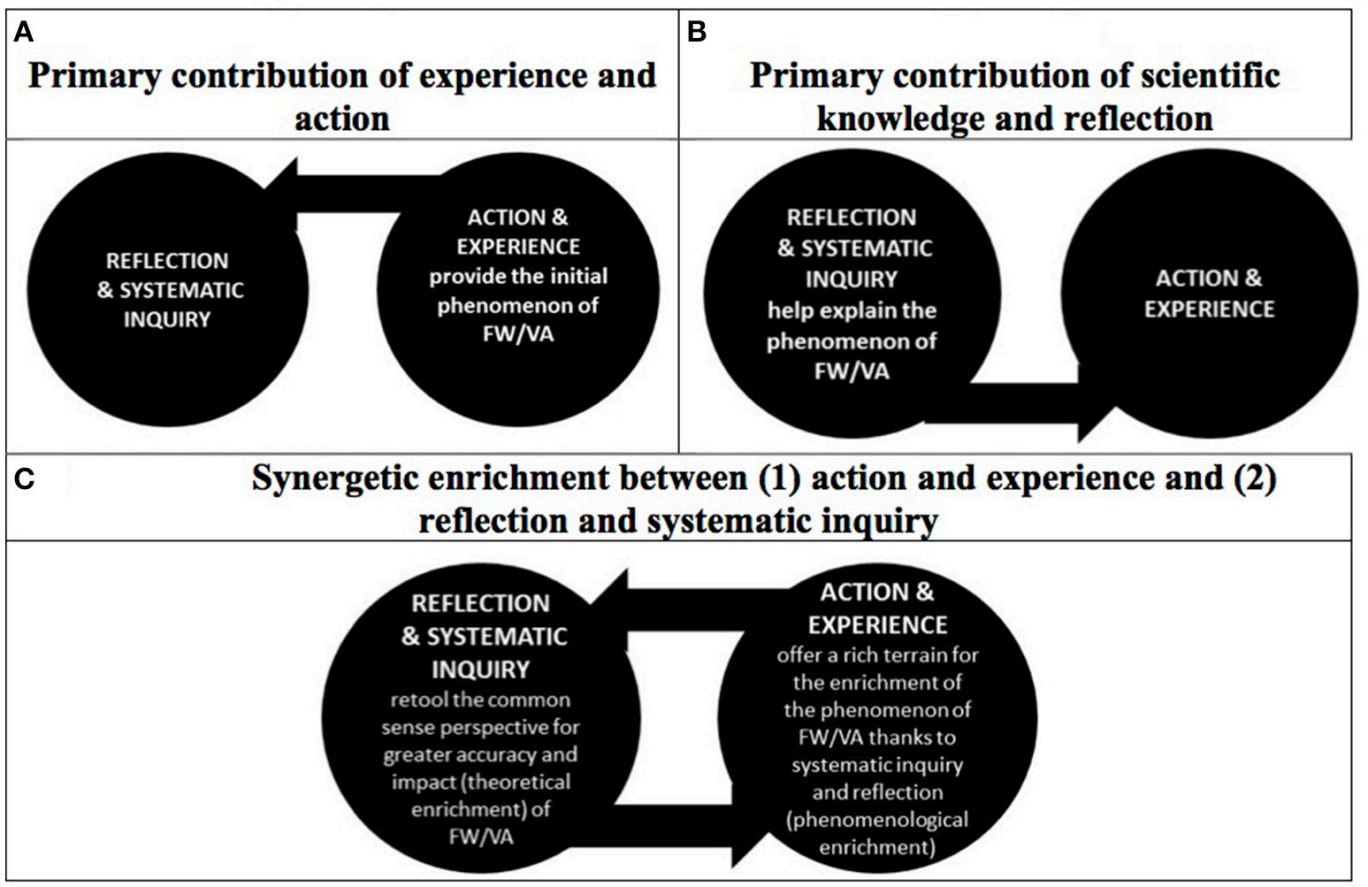
**Synergetic enrichment of the phenomenon and concept of FW/VA**. Following a dynamic epistemology inspired by pragmatism, which commands an instrumentalist method for the revision of the concept of FW/VA, scientific knowledge (broadly construed) supports a process of synergetic inquiry between (1) the phenomenon of FW/VA offered through experience and action and (2) knowledge gained leading to the refinement of the concept of FW /VA through systematic reflection and inquiry. **(A)** captures hat the action and common sense perspective is invested with the ability to provide a first person ontology to the phenomenon of FW/VA (manifest image of humans). The concept originates from an initial sense of efficacy of the will through experience and action. **(B)** captures that scientific knowledge and self-reflection (scientific image of humans) can yield new insights by, for example, reframing or re-describing first-person experiences (oneself as an object). **(C)** describes the non-dualistic, synergetic enrichment between the perspective of action and experience (phenomenological enrichment) and that of reflection and inquiry (theoretical enrichment).

Following the model of synergetic inquiries introduced above, the first-person psychological reality of FW/VA (notably its dynamic and consequential properties) needs to be captured in the concept of FW/VA but also, the first-person perspective on the phenomenon of FW/VA can be enriched by the results of scientific investigations. The broadening and increased penetrance of the resulting concept of FW/VA (and the process through which this happens) is described as “synergetic enrichment” of both the experiential phenomenon of FW/VA and the scientific concept of FW/VA. Both the first-person experience of FW/VA (e.g., as a felt moral agent, in “experience”; as an effective moral agent, in “action”) as well as the third person perspective on oneself considered as an object (via scientific knowledge) or as an objective subject (via self-reflection) can lead to synergetic enrichment of FW/VA. However, it is worth considering specifically the possible contribution of scientific knowledge to this process, which has traditionally been interpreted—based on static understandings of FW/VA—either as upholding or as invalidating the existence of FW/VA.^6^ In contrast, once the dynamic and consequential aspects of FW/VA are recognized and a dynamic epistemological framework adopted, the role of science migrates from such an ontologically revisionary contribution to a more explicitly epistemological role where synergetic enrichment between the perspectives of experience and action as well as of scientific knowledge and self-reflection can lead to a more accurate understanding of and a stronger FW/VA following an instrumentalist method for conceptual revision. Accordingly, Figure 2 captures how scientific inquiry can help sharpen and strengthen the sense of FW/VA.

First, FW/VA originates from the first person experience of the world and one's self-constructed understanding of his or her agency in the course of action (Figure 2A). Clearly, this understanding is socially constructed, shaped in interaction with others and influenced by cultural backgrounds that nourish interpretations of the phenomenon of agency. The provision of the first person ontology is the primary contribution of action and experience. Disciplines and approaches (e.g., anthropology, qualitative research) which have the ability to tap into the first person perspective and better describe its nature and scope can help provide a rich terrain for further scientific inquiry about the phenomenon of FW/VA. Recent work on the phenomenology of FW/VA has provided more traction for the first-person grounding of concepts of FW/VA suggested by pragmatist theory. Gray writes that at its most basic level, “the will is feeling in motion; it is the feeling of movement: the perceived movement of the body's internal processes or the feel of motion directed to the external world” in contrast to the feeling of inertia (Gray, 2007). As William James argues, the feeling of effort and of goal-focused activity “play a role in strengthening the internal sense of the will's capabilities” (Gray, 2007).^7^ Commenting on William James' externalist view of phenomenal or mental content (the extended mind), Krueger remarks that, “agency determines content,” i.e., there is an active construction of the phenomenal perspective based on the agent's action in contrast to a passive interpretation of the role of experience in shaping agency (Krueger, 2006). From the standpoint of pragmatist theory, it is therefore no surprise that recent cognitive science research has shown that FW/VA evolves in ways that challenge the faith in an immutable ontology since this ontology is grounded in ever-changing first-person experience and action. Furthermore, this experience of FW/VA involves other agents, which precludes a solely individualistic understanding of FW/VA and opens to intersubjectivity and a social construction of the self.^8^

Second, the third person perspective brought by self-reflection (e.g., thinking which takes the concepts describing the subject as its object such as philosophy) and scientific investigations (e.g., experimental research on FW/VA) build from an initial phenomenon of FW/VA to give meaning to this construct, and to then ground the conduct of research to a better understanding of the properties of FW/VA (Figure 2B). Self-reflection and critical conceptual analyses can help sharpen the concepts used to describe FW/VA while experimental research can test how FW/VA works in the complex reality of varying circumstances (e.g., effects of personality, socio-economic contexts, gender, pathologies). For example, research has suggested that patients with schizophrenia may have specific deficits leading to the dissociation between intents and authorship of actions. In other words, they may not claim the authorship for their actions and they may attribute intents to non-effective moral agents (Lafargue and Franck, 2009).

Finally, the perspective of action and experience gives a first ontology and meaning to FW/VA but is then in a possible synergetically-enriching relationship with research and reflection following a non-dualistic relationship between the first person perspective of action and the third person perspective of reflection and inquiry (Figure 2C). This analysis avoids the dead-ends produced by the dogmatism of believing that only one of the constitutive perspectives (first person experience or third person inquiry) provides a foundational stance on FW/VA in ways that justify the reduction of FW/VA to one element (first person phenomenon or the object of third person inquiry). For example, realists who oppose any possible revision based on scientific research (Morse, 2007), refuse to recognize the ability of science to provide insights into the understanding of FW/VA. In contrast, the scientific eliminativists uphold that only science can provide a legitimate perspective, thereby not acknowledging the origins of the concept and its grounding in first person ontology. The extreme eliminativists would even absurdly propose to eliminate the concept of FW/VA (Haggard, 2011) thus confusing the experience of FW/VA (the ontology, originating from the first person perspective) with our knowledge of FW/VA (the refined epistemology provided by science). The proposed synergetic model of inquiry also suggests how the first person and third person perspectives may interact. Scientific research and self-reflection can provide more accurate tools for the exercise of the will while the first person ontology furnishes science with the phenomenon of will and voluntary action. The overall promise is a better understanding of FW/VA and a promise of its greater power and efficacy in human affairs. Thus, rather than being disconfirmed or discounted by neuroscience, the understanding of FW/VA can actually be enriched and refurnished. As Dewey writes, a better understanding of the mechanisms of agency can empower moral agents:

“We are told that seriously to import empirical facts into morals is equivalent to an abrogation of freedom. Facts and laws mean necessity we are told. The way to freedom is to turn our back upon them and take flight to a separate ideal realm. Even if the flight could be successfully accomplished, the efficacy of the prescription may be doubted. For we need freedom in and among actual events, not apart from them” (Dewey, 1922).

My use of the terminology of “mind-brain” here and elsewhere captures that scientific inquiry is not the sole appanage of the biological sciences (Racine, 2010); the humanities and social sciences bring important contributions to the understanding of FW/VA. Again such a view is consistent with pragmatist philosophy and its stance toward the contribution of neuroscience (and science more generally) to the understanding of folk psychological concepts such as FW/VA. Dewey proposes that basically all mind-concepts are susceptible to revision based on scientific inquiry; these concepts are considered fluid self-interpretations influenced by factors such as social contexts, learning, experience, and culture. However, for Dewey these “epitomes” which stem from experience and action are not arbitrary, even if they do not originate from scientific inquiry, since they are legitimate experience-based efforts to self-understanding grounded in the manifest image of humans (Sellars, 1963). Accordingly, fluidity and approximation in self-understanding also implicate differing ways in which FW (or more concretely VA) is understood. In pragmatist theory, a key task of philosophy and science is to deconstruct the implied assumptions found in common sense concepts through criticism and experimental research to then refurnish them (or related concepts) with enriched meaning based on inquiry. However, as Dewey warns, contrary to the sweeping reductionism of the scientist-eliminativist neurophilosophy, “it will be a long time before anything of this sort will be accomplished for human beings. To expel traditional meanings and replace them by ideas that are products of controlled inquiries is a slow and painful process” (Dewey, 1922).^9^^,^^10^

The model proposed here and inspired by pragmatist philosophy helps clarify why the position of the reductionists and eliminativists can be seriously questioned. On the one hand, reductionists and eliminativists attribute the power of science to settle ontological questions about what *is*, i.e., the object of science is established by science, which brings in circular reasoning. On the contrary, the initial ontology of FW/VA comes from the first person perspective, thus creating a performative contradiction for strong reductionism and eliminativism. A more moderate interpretation consistent with our analysis can accommodate a revisionary role for science in vetting presumed ontological entities which have no bearing in reality (e.g., wrong attributions of FW/VA to individuals suffering from severe deficits, acknowledging modulations to FW/VA based on priming and contextual effects). A moderate form of revisionism is also consistent with the bulk of emerging research which recognizes the existence of FW/VA in first-person accounts as well as the socially-constructed nature of concepts of FW/VA (Baumeister, 2008; Stillman et al., 2011; Baumeister and Monroe, 2014). As an enterprise to understand the world, scientific inquiry also reveals some aspects of reality that are otherwise concealed from the standpoint of common sense (i.e., without the support of scientific inquiry). Therefore, neuroscience has both an epistemic and an ontological contribution to make, although it is likely that the contribution of the latter is much more subtle than what is assumed by most eliminativists.

On the other hand, realists refuse in principle the possible insights of science and believe in a static ontology (or they fail to capture the possibility of science to support the theoretical enrichment of the concept of FW/VA). One possible argument is that—akin to those encountered in the first wave of research described above—such research has no implications because it is either too complex to take into account (generates a cognitive over-load) or impractical (i.e., categories for FW and VA need to be clear cut to support the determination of responsibility in law and ethics). The impact of the transition from a general static metaphysics to a dynamic epistemology would thus carry a challenging potential implication. This is, namely, that when one refers to voluntary action and to free will, he or she is alluding to a highly plastic and ever-changing cognitive and behavioral phenomenon that lacks the typical (and convenient) stability attributed to a general essentialist concept of FW/VA (Table 2). It is important to reassure that some core features of FW are likely well-captured in lay intuitions (e.g., ability to make a choice, acting consistently with one's desires, being reasonably free of constraints (Stillman et al., 2011; Baumeister and Monroe, 2014) but its contours may be unclearly defined and changing, notably based on refined scientific understandings of the phenomenon of FW/VA. Consequently, it would be puzzling to rule out the contribution of research, and thus affirm that scientific inquiry about FW/VA has none and will never have any practical implications whatsoever.^11^ In addition, there is no need to interpret the contribution of the scientific perspective as a complete revolution (only an evolution) from previous understandings of FW/VA. Thus, an instrumentalist analysis of the concept of FW/VA grants neuroscience and cognitive neuroscience an epistemological contribution via the process of synergetic enrichment. One immediate outcome of neuroscience is the catharsis of expelling from our self-understanding ideas about FW/VA which are partially or completely erroneous. But there is also a promise that a more accurate understanding of FW/VA will help human beings better understand their and other's behaviors and perhaps better direct it or support it to channel human behavior to desired goals, which brings us to implications of this revised understanding of FW/VA.

### Possible concrete implications of the instrumentalist analysis of the concept of FW/VA and of synergetic models of inquiry

Following an instrumentalist analysis of the concept of FW/VA and the model of synergetic models of inquiry, some methodological and theoretical implications for research as well as implications for practice can be foreseen at this time, as much as this can be envisioned given gaps in the current state of research on FW (Ewusi-Boisvert and Racine, under review).

#### Implications for research

Concrete implications for research follow from the proposed model of synergetic enrichment stemming from instrumentalism (Figure 2C). **(1) No a priori commitment to eliminativism**. Research on the nature of FW/VA should be carried out without strong initial biases for or against the ability of the concept of FW/VA to capture folk-psychological perspectives in real life and real world settings because both the first person and third person perspectives are needed and they can also co-evolve. Interesting work has now been initiated in this direction although the stability and validity of findings should be confirmed through replication (including cross-cultural studies) and greater sensitivity to the context in which the data are collected (e.g., simple tasks and prompts vs. more complex—ecologically plausible—tasks and prompts; Ewusi-Boisvert and Racine, under review). The requirement of understanding the subjective complexity of FW/VA should, in principle, help provide a richer terrain for the experimental investigations that try to tease apart the mechanisms involved in FW/VA as well as how these mechanisms are associated with other behavioral and psychological phenomena. Recent research acknowledges the necessity of capturing this reality because of the socially constructed nature of the phenomenon of FW/VA (Nahmias et al., 2004, 2014). Otherwise said, the first person ontology of FW/VA has to be fully acknowledged and taken seriously scientifically to help neuroscience research build from it (not eliminate it; Brass et al., 2013). Therefore, disciplines that can offer to flesh out the diversity and richness of folk concepts of FW/VA can help ground investigations in actual life world events and experience.

**(2) No a priori commitments to the epistemic supremacy of neuroscience or of lived experience**. The proposed instrumentalist analysis of the concept of FW/VA (Table 2; Figures 1, 2) calls for a genuine contribution of disciplines that shed light on the action-experience component of FW/VA and its origins in agency. Currently, FW is often operationalized in experimental research based on its simplest expression such as the conscious initiation of motor behavior (Dubljević et al., 2014). This is a necessity of experimental research which attempts to start with simpler and tractable problems and move to more complex ones. However, in common interpretations, the limited scope of this research has not always been recognized. A systematic review of the studies which followed the landmark Libet experiments has shown that unfortunate sweeping interpretations were generated much beyond the initial limited scope of these experimental studies and their significant limitations (Dubljević et al., 2014).^12^ It is quite apparent that an understanding of FW/VA in its full complexity requires much more sensitivity to the effects of context and experimental procedures. For example, common scales used to operationalize beliefs in FW/VA could be more sensitive to the actual understanding of FW/VA in the social world (Paulhus and Carey, 2011). It would be advisable to reduce blind faith in the epistemic supremacy of either biological perspectives or those of the social sciences and humanities; they bring different enrichments to the complex and multifaceted concept of FW/VA. Eventually, interdisciplinary scholarship or dialogue will perhaps be crucial to reap the benefits of the knowledge gained. Accordingly, foundational perspectives, both of action/experience (or common sense) perspectives or of scientific perspectives (especially in the forms of biological essentialism and reductionism), as described above, should be avoided. Elimination should be admitted on a piecemeal basis, i.e., once an aspect of FW/VA is determined to be inexistent, such a “false positive” can be ruled out. Thus, the ontology of FW/VA can be revised but this should occur thoughtfully and without a priori commitments to overriding foundational stances.

**(3) Careful translation of research on FW/VA**. Practical and agential issues associated with research on FW/VA should be considered in their own rights, which neither means completely separately nor dichotomously from theoretical issues. However, early findings and observations should be respected for what they are, i.e., as belonging initially to the domain of scientific inquiry and reflection. The route to the refinement of concepts describing FW/VA could be long and pressures to hastily draw conclusions about matters such as responsibility have imperiled knowledge gains (Racine et al., 2016). Timely public communication of research results should avoid disrupting concepts of FW/VA with important social functions without being able to provide a knowledge-based, synergetically enriched, concept (or concepts) of FW/VA. Indeed, the default buttress should be an existing and commonly shared concept of FW/VA (even if imperfect) which has traction in experience and action instead of cavaliering to a premature and allegedly theoretically enriched concept.

#### Implications for practice

At this stage and with the level of evidence gathered thus far on FW/VA (Ewusi-Boisvert and Racine, under review), the practical implications of the latest wave of research on FW/VA cannot be fully fleshed out in detail. Furthermore, their actual translation in practice would also be premature given the level of evidence supporting some of the key observations. However, reflection on how a significantly revised concept of FW/VA could so inform practices—if current conclusions about the dynamic and consequential aspects of FW/VA gain strong replication and broader generalization in larger study samples—is worth initiating (see Table 3).^13^

**Table 3:** **Examples of possible practical implications of an instrumentalist and synergetically-enriched concept of FW/VA**.

**Characteristics of an instrumentalist account of FW/VA (see Table 2)**	**Implications for…**	
	**Self-understanding and self-image**	**Social interactions and social life**
Approximate, umbrella concept	Reveal cognitive biases in one's own style or that of others in conceptual categories related to FW/VA. Reveal blind spots, i.e., incongruences between one's (implicit) experience and understanding of FW/VA and the actual more global phenomenon to be captured as well as one's entertained explicit concept of FW/VA.	Explore the use of support systems/technology to assess levels of voluntariness by taking into account modulatory factors and mitigating circumstances. Current use of human intelligence alone may not pay justice to the knowledge gained about FW/VA through scientific inquiry. The cognitive constraints of moral agents may prevent the factoring in of multiple aspects—this is likely acceptable for common everyday action but deficient for cases where actual responsibility needs to be determined and has serious implications.
Dynamic & scaled on a continuum, can increase or decrease	Recognizes the evolving refinement of the understanding of FW/VA—develop dynamic epistemology in ethics that grants for such insights. Recognize the impact of context and specific circumstances in the development of an understanding of (increased/stable/diminished) FW/VA (e.g., understanding VA in a simple motor task may be very different (socially and biologically) than FW/VA in social life. Additionally, one's attribution of FW/VA could be biased by a plethora of factors (e.g., implicit attitudes toward gender, racial group), i.e., positional biases.	Revise the application of the concept of VA in law; integrate the context-induced fluidity of FW/VA in judgments about responsibility (e.g., if certain factors are known to diminish or imperil FW/VA and to what extent they are). Develop discourse with a greater understanding/recognition of the biases based on scientific research. Reconsider how different socio-cultural contexts lead to different frameworks with respect to the understanding of FW/VA (are similar intents, gestures, etc. considered equally voluntary in different socio-cultural contexts, and why).
Originates from one's own sense of agency but the understanding of which can be refined through scientific inquiry.	Reveal the perspectival/contextual (as they relate to interpretations of the agent) nature of constructs about VA based on a potential range of experiences and social determinants to investigate (e.g., impact of socio-economic status, gender, religion, age).	Refurnish the meaning of concepts of FW/VA based on a more scientific understanding of agency and of voluntary action, eventually taking into account the impact of life experience and social determinants.

## Conclusion

La science est l'asymptote de la vérité. Elle approche sans cesse et ne touche jamais.

—Victor Hugo, William Shakespeare^14^

Debates about the philosophical and ethical implications of empirical research about FW/VA have centered on ontological implications (existence or non-existence of FW/VA), which represent largely metaphysical debates. Potentially more interesting and difficult to integrate are recent studies suggesting that the phenomenon FW/VA has dynamic and consequential properties. In general, this research suggests that beyond the debates about the actual existence of FW/VA, there is a great need to explore how our understanding of this complex phenomenon could be enriched and revised to take into account recent research. This is what I have tried to capture following an instrumentalist account of the concept of FW/VA which grants dynamic and consequential aspects to FW/VA. These properties make the case for the role of both the action/experience perspective as well as the contribution of scientific inquiry to the enrichment of our understanding of FW, provided that some methodological and conceptual pitfalls are avoided. It is likely that the implications of this research will be modest (less metaphysical; more epistemological) in scope than some prior discussion about the implications of neuroscience for FW. They may also require extensive replication. It is early to predict the definitive implications of this research but I have proposed that various forms of positional biases and blind spots could be revealed to nurture reflection and increased self-understanding of moral agents with respect to the understanding of FW/VA. Whether such epistemological implications would morph into more substantive ethical and policy implications is contingent on the negotiation between experience-based understandings of FW/VA and the enriched meanings brought forth by research. Indeed, I have suggested that, taken as a practical concept of daily life, FW/VA initially finds meaning in action and experience, and there should be no presumptuous preclusion of how research will evolve and what will be learnt about how FW/VA concepts adequately-inadequately describe this complex phenomenon.

## Author contributions

The author confirms being the sole contributor of this work and approved it for publication.

## Funding

The writing of this article was supported by a grant from the Social Sciences and Humanities Research Council of Canada held by ER (# 410-2011-1606) as well as a career award from the Fonds de recherche du Québec—Santé (#30998).

### Conflict of interest statement

The author declares that the research was conducted in the absence of any commercial or financial relationships that could be construed as a potential conflict of interest.

## Footnotes

^1^Since free will is essentially defined as an ability to make decisions and complete actions, I abbreviate this concept as FW/VA following the philosophical analysis of Gert and Duggan (1979). I also sometimes use the separate abbreviations of FW and VA to respect the intent of other authors.

^2^Note to readers: I distinguish the “phenomenon of FW” from the “concept of FW” in the following way: (1) the phenomenon of FW designates the first-person experience of having the ability to make free decisions and act upon them while the “concept of FW” describes the scientific and philosophical construct that takes the phenomenon of FW as its objects. It is clear that the phenomenon of FW is not only subjective (but intersubjective) and embedded in an individual's thinking about the world, and could thus be considered a concept. I could have therefore used the term of “psychological concept of FW” to designate this reality but this would have sowed confusion if, in contrast, I would have named, for example, the “concept of FW,” the “scientific concept of FW” since one could have interpreted my writing has setting an opposition between psychology and science which is contrary to my intents. This distinction also reinforces that the concept of FW is an instrument to capture the phenomenon of FW and is therefore derivative of that phenomenon which is here considered as intrinsically subjective and intersubjective.

^3^For a review of the possible relationships between both areas, see the review paper by Brass et al. (2013).

^4^The charged term of “FW” has provoked debates about whether an action is initiated solely by the moral agent (i.e., whether the will of the moral agent needs to be entirely undetermined by external causes). Clearly, such a strong interpretation is problematic because it implies that the will must be a “free will,” subtracted from the forces of causality (i.e., an “uncaused causer”). The interpretation then begs the question of how the will can be considered as a force that can cause actions and events without being part of the order of other phenomena which follow causal chains.

^5^A recent literature has shed further light on the role of inhibition in flexible behaviors in humans. The theoretical proposal is that the choice of a specific goal-directed action is contingent on its probability of yielding the best outcomes, here defined in terms of biological fitness (Mirabella, 2014). Two distinct executive functions could be at stake in generating such flexible behaviors: “(i) the ability to predict future outcomes of goal-directed actions; and (ii) the ability to cancel them when they are unlikely to accomplish valuable results” (Mirabella, 2014). Importantly, those two functions could be scaled on a spectrum and rely on overlapping brain regions involved and “intimately linked (directly or indirectly) to the evaluations of pros and cons of an action” (Mirabella, 2014). Experimental evidence supporting this theory has examined the neuronal basis of inhibition of movement in non-human primates (Mirabella et al., 2011; Mattia et al., 2013), in Parkinson's patients treated with deep brain stimulation and in pharmaco-resistant epileptic patients, which offer human models to explore the role of certain pathways in inhibition (Mattia et al., 2012; Mirabella et al., 2012), and also using transcranial magnetic stimulation (Coxon et al., 2006). It is possible that an instrumentalist understanding of FW/VA as a dynamic and consequential psychological phenomenon could be consistent with this understanding of the neural mechanisms that grant flexibility to human behavior.

^6^Sellars describes this tension as the conflict between the “scientific image” and the “manifest image of” of humans (man) (Sellars, 1963). I capture this tension between the (1) perspective of action and common sense (or folk psychology) which requires applicability, relevance, and appropriateness (practical criteria) for FW/VA and the (2) reflection and scientific inquiry where the highest level of conceptual clarity and scientific evidence is pursued (epistemological criteria).

^7^From Dewey: “By will, common-sense understands something practical and moving. It understands the body of habits, of active dispositions which makes a man do what he does. Will is thus not something opposed to consequences or severed from them. It is a *cause* of consequences; it is causation in its personal aspect, the aspect immediately preceding action. It hardly seems conceivable to practical sense that by will is meant something which can be complete without reference to deeds prompted and results occasioned.” (Dewey, 1922).

^8^Dewey writes about common invidualistic and reductionistic views that: “There are specific good reasons for the usual attribution of acts to the person from whom they immediately proceed. But to convert this special reference into a belief of exclusive ownership is as misleading as to suppose that breathing and digesting are complete within the human body.” (Dewey, 1922).

^9^Dewey was, in this case, comparing research on human nature to the physical sciences in which technical language is vested with new insights and power because of the research supporting conceptual refinements.

^10^Daniel Dennett has revitalized part of Dewey's “instrumentalism” in philosophy of mind through the theory of the intentional stance. This is the thesis that the attribution of folk psychological concepts (such as intentions and beliefs) plays a useful role in the explanation of behavior and psychology (Dennett, 1981) although it has been argued to be amenable to scientific inquiry and revisions (Racine, 2005). Dennett is also one of the few who has hinted to the folk psychological and instrumental role that the concept of FW plays, helping to capture and interpret our own sense of agency and explain how we are causally efficacious (or inefficacious) agents in our personal and social lives (Racine, 2005).

^11^An interesting counter-example lies in the practical interventions surrounding the raising of awareness about implicit attitudes in legal judgments (National Center for State Courts, 2012). The US National Centre for State Courts produced a discussion document to capture how legal reasoning could be amenable to greater awareness and specific interventions for de-biasing based on recent psychosocial research. There are potentially similar circumstances where a fine grain understanding of FW/VA could be useful to help provide more accurate assessments of the “willed” or voluntary nature of behaviors (and recognition of the factors modulating perceptions thereof).

^12^A subsequent systematic analysis of media portrayal of this study has shown a similar phenomenon (Racine et al., 2016).

^13^The writings of Dewey on ethics stage the current tension at hand between his original theses of “radical empiricism” in ethics and of “presumed (or expected) responsibility” in an interesting way. From the scientific perspective, there is a potential to enrich our understanding of FW/VA and their relationship to agency and responsibility (e.g., how they are conceived and the conditions for their proper attribution). However, on the other hand, the threat to presumptions of agency and responsibility posed by research on FW/VA seem unsettling to the point where the wisest solution could be, for now and from a practical standpoint, to put in bracket the perspectives that science is currently offering (e.g., limited data, limited replication, lack of ecological validity of study designs) until stronger evidence is gathered. Some authors make the case for a preserved (but non-metaphysical) belief in FW/VA, notably because of its role in legal affairs and democratic life (Morse, 2006; Dubljević, 2013). The tension between the theses of radical empiricism and default responsibility also points to the tension between how science challenges and refines our self-understanding even though we entertain commonsensical perceptions and understandings of how we are based on practical need, conventions, habits, etc.

^14^“Science is the asymptote of truth; it approaches unceasingly, and never touches.” (Hugo, 1887)
